# KRT80 Promotes Lung Adenocarcinoma Progression and Serves as a Substrate for VCP

**DOI:** 10.7150/jca.91753

**Published:** 2024-02-25

**Authors:** Shanhua Huang, Weilai Tong, Bowen Yang, Li Ma, Jiaming Zhang, Chunliang Wang, Linlin Xu, Jinhong Mei

**Affiliations:** 1 Department of Pathology, The First Affiliated Hospital, Jiangxi Medical College, Nanchang University, Nanchang, China.; 2 Institute of Molecular Pathology, Jiangxi Medical College, Nanchang University, Nanchang, China.; 3 Department of Orthopedics, The First Affiliated Hospital, Jiangxi Medical College, Nanchang University, Nanchang, China.; 4 Department of Neurosurgery, The First Affiliated Hospital, Jiangxi Medical College, Nanchang University, Nanchang, China.

**Keywords:** lung adenocarcinoma, KRT80, VCP, PI3K/AKT pathway, progression

## Abstract

**Background:** Keratin 80(KRT80) encodes a type II intermediate filament protein, known for maintaining cell integrity of cells and its involvement in the tumorigenesis and progression of various cancers. However, comprehensive research on its relevance to lung adenocarcinoma remains limited.

**Methods:** In this study, we utilized multiple databases to investigate the transcriptional expression of KRT80 and its correlation with clinicopathological features. A range of assays, including the Cell Counting Kit 8 assay, colony formation assay, cell migration assay, and flow cytometry, were employed to elucidate the impact of KRT80 on the malignant behavior of lung adenocarcinoma. Immunoprecipitation and mass spectrometry were also used to identify putative genes interacting with KRT80.

**Results:** The expression of KRT80 was elevated in lung adenocarcinoma and patients with high levels of KRT80 expression had poor clinical outcomes. Silencing KRT80 suppressed cell viability, and migration, while overexpression had the opposite effect. In addition, Immunoprecipitation and mass spectrometry revealed an interaction between KRT80 and valosin-containing protein (VCP), with VCP knockdown reducing the stability of KRT80 protein. Overexpression of KRT80 mitigated the inhibitory effect of VCP knockdown to some extent.

**Conclusion:** Our findings collectively suggest that KRT80 is a promising diagnostic and prognostic indicator for lung adenocarcinoma. Additionally, the interaction between KRT80 and VCP plays a crucial role in the progression of lung adenocarcinoma, which implies that KRT80 is a promising therapeutic target.

## Introduction

Lung cancer remains a formidable global health challenge, as estimated by the GLOBOCAN project, with approximately 2.20 million newly diagnosed cases worldwide, constituting 11.4% of all new cancer diagnoses. Notably, lung cancer remains the leading cause of cancer-related deaths, accounting for 18.4% of all cancer fatalities 1. In the United States, approximately one-quarter of all cancer-related deaths can be attributed to lung cancer 2. Among lung cancer cases, 85% are classified as non-small cell lung cancer (NSCLC), with lung adenocarcinoma (LUAD) being the predominant subtype 3. In recent years, targeted therapy and immunotherapy have led to a promising increase in survival rates, with a 5% to 10% improvement observed in 21 countries between 1995-1999 and 2000-2014^4^. However, despite these advances, the overall 5-year survival rate for lung cancer ranges from 68% in Stage IB to a mere 0%-10% in the advanced stages of IVA-IVB 5. This grim prognosis is compounded by the fact that 75% of lung cancer cases are diagnosed at the advanced stage IV 4, 6.

The cell's structural framework, the cytoskeleton, plays a pivotal role in maintaining cell morphology, division, differentiation, motility, and adhesiveness. The cytoskeleton consists mainly of microtubules, actin, and intermediate filaments (IFs) 7. Among Ifs, six categories exist, including type I acidic keratins and type II keratins. Human type I epithelial keratin composes 17 types: K9, K10, K12-K20, and K23-K28, while human type II epithelial keratin includes 18 types: K1-K8 and K71-K80 8. KRT80 is an IF-forming protein in epithelial cells with a molecular weight of 50.5 kDa, composed of 452 amino acids. Its expression product is integral to cytoskeletal assembly and preserving epithelial cell integrity 9. In several tumor types, KRT80 is upregulated, contributing to malignant oncological behaviors, such as those seen in colon cancer, gastric cancer, ovarian cancer, and breast cancer 9-14. These findings suggest KRT80's potential as a therapeutic target and a valuable diagnostic and prognostic indicator. Nevertheless, the extent of comprehensive research on KRT80's role in LUAD is limited.

VCP belongs to the ATPases associated with various cellular activities (AAA) family characterized by four distinct domains: the N-terminus for substrate recognition and cofactors interaction, the C-terminus domain for nuclear localization, and two ATPase domains. The D1 domain participates in oligomerization and hexamer assembly, while the D2 domain catalyzes ATP hydrolysis. VCP's diverse set of cofactors allows it to recruit proteins to various cellular locations, contributing to a range of processes, including chromosomal, ribosomal, mitochondrial, endoplasmic reticulum-related degradation (ERAD), autophagy, and protease gene expression, apoptosis and cell cycle regulation 15. Multiple VCP cofactors contain ubiquitin-regulated X(UBX) and UBXL (UBX-like) domains, suggesting a potential connection to ubiquitination processes. Aberrant upregulated of VCP has been observed in various cancers, promoting cell migration and invasion in colon cancer, liver cancer, stomach cancer, breast cancer, lung cancer, and glioma 16. However, research on VCP's ubiquitination in the context of cancer remains limited.

In our study, we employed a multifaceted approach, including bioinformatics analysis, clinical samples, vitro experiments, and immunoprecipitation assays to provide a comprehensive understanding of KRT80's role in LUAD and its underlying mechanisms. For the first time, we unveil the interaction between KRT80 and VCP, shedding light on its carcinogenic impact through the activation of the PI3K/AKT pathway.

## Methods

### Source and processing of bioinformatics analysis

The expression of KRT80 in cell lines and the pan-cancer analysis of KRT80 were obtained from The Human Protein Atlas and TCGA, respectively. We investigated the relationship between KRT80's mRNA expression level and its gene DNA methylation level on the cBioportal. Ferroptosis-related genes were acquired from FerrDb. Transcriptome data, comprising 57 LUAD patients and 11 normal lung tissues were extracted from the GEO database. Additionally, we obtained transcriptome data from 59 normal lung samples and 539 LUAD tissues, along with their matching clinical information from TCGA database. The clinical information of 539 LUAD patients was presented in Table 1. Among these, 494 cases of LUAD with prognostic data and the detailed dataset were included in the Supplementary Table 1. In correlation analysis, genes with |Cor| >0.3 and an adjusted p-value<0.001 were defined as KRT80-related genes and detail data was presented in Supplementary Table 2.

### Tissue samples and clinical data

This study was approved by the Ethics Committee of the First Affiliated Hospital of Nanchang University. We collected paraffin-embedded LUAD cancer tissues with confirmed diagnosis and eight pairs of clinical tissues. Patients who had received preoperative chemotherapy or chemoradiotherapy were excluded from this study. The clinical features of 115 paraffin-embedded LUAD cancer tissues were presented in Supplementary Table 3.

### RT-qPCR and Western blot

RNA extraction was performed using the Vazyme RNA extraction kit, following the provided instructions. HiScript II Q RT SuperMix for qPCR (+gDNA wiper) (Vazyme, China) was used to generate cDNA, and ChamQ Universal SYBR qPCR Master Mix (Vazyme, China) was employed to measure the mRNA expression of target genes in cells and tissues. The following primer sequences was used for KRT80:

Forward: 5'-CTGGAGAGCTTCGTGGAGTTGATG-3'

Reverse: 5'-GTCACCGACACATCCTTCACCTG-3'.

Cell lysates containing 1% protease inhibitors were used for protein analysis. Then, the same amounts of proteins were used for electrophoresis in 7.5% SDS-PAGE and then transferred to the PVDF membrane. The membrane was blocked with 5% milk and then incubated overnight with various primary antibodies. The primary antibodies used included GAPDH (60004-1-Ig, Proteintech), KRT80 (16835-1-AP, Proteintech), VCP (PB9454, Boster), HA-tag (HRP-66006, Proteintech), Myc-tag (2276S, CST), PI3K (4249, CST), P-PI3K (4228, CST), AKT (60203-2-Ig, Proteintech), and P-AKT (66444-1-Ig, Proteintech), PCNA (10205-2-AP, Proteintech), CyclinD1 (26939-1-AP, Proteintech), MMP9 (AF5228, Affinity), N-cadherin (22018-1-AP, Proteintech), Vimentin (10366-1-AP, Proteintech), E-cadherin (20874-1-AP, Proteintech). Then a secondary antibody was applied for 1 hour.

### Cell culture and transfection

We obtained LUAD cell lines (NCI-H1299, A549, NCI-H1650, HCC-827, PC9, NCI-1975 and BEAS-2B) from the Laboratory of the First Affiliated Hospital of Nanchang University. Cells were cultured in a complete medium at 37°C and 5% CO_2_. Transfections were performed using the Turbofect transfection reagent (Invitrogen, USA). Stable cell lines were established through transfection with pLV3-U6-VCP (human)-shRNA1-CopGFP-Puro, followed by selection with puromycin (1μg/ml). PCMV-KRT80 (human)-3×HA-Neo and pCMV-VCP (human)-3×Myc Neo were transfected into 293T cells for co-immunoprecipitation. Various small interfering RNAs and plasmids were transfected into LUAD cell lines. The target sequences for KRT80-siRNA and VCP-siRNA were as follows:

KRT80-Homo-123: GCUCCUGCGUGGUUGGCUUTT.

KRT80-Homo-258: GCUGGUCGGCUGGCACUAUTT.

KRT80-Homo-685: CCUGGAUGCAGAGUGUCUUTT.

VCP-homo-2027: GCUGCUCACCAUGUGGUUUTT.

VCP-homo-588: GCUGUUUGCAUCGUCCUUUTT.

### Immunohistochemistry

Paraffin-embedded tissue samples were cut into 4 μm sections, followed by antigen retrieval with EDTA. Endogenous peroxidase was blocked using 3% hydrogen peroxide, and the sections were incubated overnight with primary antibodies. After primary antibody incubation, a second antibody was applied for 15 minutes at 37°C before DAB staining. KRT80 staining was scored based on the product of staining intensity and staining area. With the following criteria: Intensity standards: 0 (-), 1 (+), 2 (++), and 3 (+++). Staining area standards: <25%=1, 25%-49%=2, 50%-74%=3, >75%=4. Samples with scores greater than 6 were classified as the high- expression.

### Cell proliferation experiment

Cells transfected for 48 hours were inoculated into 96-well plates, at a density of 2000 cells per well. The time when cells were fully adhered was considered 0 hours. Cell Counting Kit 8 reagent was added to the complete culture medium at 0, 24, 48, and 72 hours after cell adhesion, OD values were measured after incubation at 37°C for 2 hours. The cell cloning experiment involved cultivating 2000 transfected cells in a 6-well plate. After 2 weeks, cells were fixed with methanol and stained with crystal violet.

### Migration assay and flow cytometry analysis of cell cycle

In the migration assay, a high-concentration medium was placed in the lower chamber, and 70,000 cells in serum-free medium were suspended in 200ul and added to the upper chamber. Following incubation, cells were fixed and stained with methanol and crystal violet. The number of migrated cells was quantified using ImageJ. For cell cycle analysis, cells were digested, washed with cold PBS, and fixed with 70% ethanol stored at -20°C. After fixation at 4°C for 2 hours, cells were stained using the Working Solution (C543, Dojindo), and analyzed using flow cytometry.

### Co-immunoprecipitation assays and Protein stability experiment

In co-IP experiments, the primary antibody was added to protein lysate and incubated overnight. The lysate was then mixed with protein A/G magnetic beads (HY-K0202, MedChemExpress) for 2 hours, and the beads were subsequently resuspended with 2X SDS sample buffer. For protein stability experiments, Cycloheximide (CHX, 50μg/mL, MedChemExpres) and/or MG132(20μM, HY-13259, MedChemExpress) were added to the corresponding groups.

### Immunofluorescence

Immunofluorescence involved the fixation and permeabilization of cells successively with paraformaldehyde and Triton X-100. Cells were then incubated with antibodies against two target proteins 16 hours, followed by incubation with fluorescent secondary antibodies for 1 hour. Nuclear staining was performed using DAPI, and images were acquired using a confocal microscope.

### Statistical analysis

Data was analyzed using R software (version 4.1.2), ImageJ, SPSS (version 23.0), and GraphPad Prism 8.3. Differences between two independent groups, and paired groups were assessed using the t-test or Mann-Whitney test, while comparisons among three groups were conducted using parametric or Kruskal Wallis test. Kaplan-Meier analysis was used to visualize the impact of KRT80 expression on survival, with differences assessed by the log-rank test.

## Results

### Aberrantly high expression of KRT80 in LUAD has a worse prognosis based on analysis of public databases

**Figure 1:**
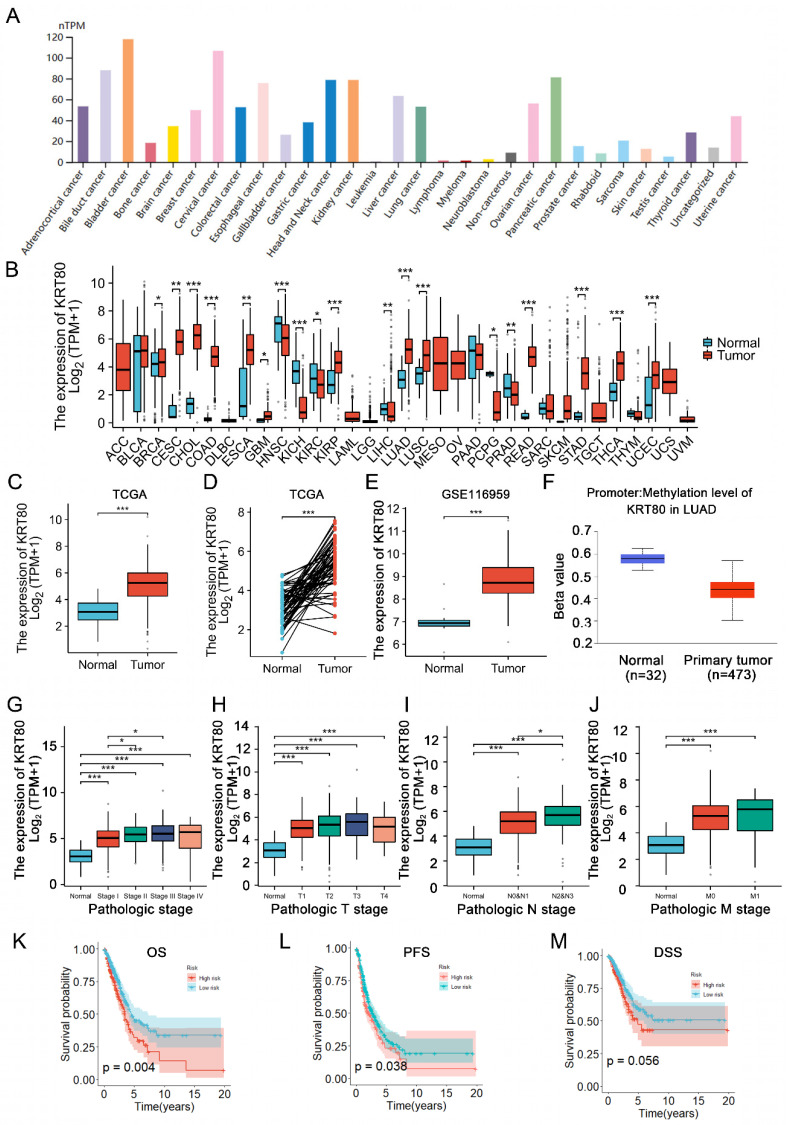
KRT80 expression status in tumors. **(A)** KRT80 expression in tumor cell lines. **(B)** Expression levels of KRT80 in various tumors from the TIMER database. **(C-E)** KRT80 expression in LUAD tissues compared to unpaired or paired paracancerous tissues in the TCGA database and GEO database. **(F)** Correlation of KRT80 mRNA levels with its DNA methylation analyzed in the cBioPortal database. **(G-J)** Expression of KRT80 in subgroups. **(K-M)** Kaplan-Meier curves for Overall Survival (OS), Progression-Free Survival (PFS), Disease-Specific Survival (DSS), and Progression-Free Interval (PFI) in LUAD. *P < 0.05, **P < 0.01, ***P <0.001, ****P < 0.0001.

Analysis of KRT80 expression across various tumors using the TIMER2 database, revealed differential expression in 18 cancers compared to adjacent normal tissue among 22 tumors. KRT80 was found to be upregulated in 13 tumors, including LUAD (**Figure 1A, B**). Consistent with these findings, data from TCGA and GEO databases demonstrated elevated KRT80 expression in LUAD (**Figure 1C-E**). We observed that DNA hypomethylation of KRT80 might account for its high expression (**Figure 1F**). Furthermore, stratified analysis of patient data from the TCGA database showed that the expression level of KRT gradually increased with the progression of the disease, which suggesting that KRT80 may be involved in the deterioration of LUAD (**Figure 1G-J**). Analysis of patient data from the TCGA database indicated correlations between KRT80 expression levels and various clinical features, such as gender (p=0.035), pathological clinical staging (p=0.002), and T (p=0.024) and N staging (p=0.001) (**Table 2**). Survival analysis based on multiple outcome indicators, including overall survival (OS) (**Figure 1K**), progression-free survival (PFS) (**Figure 1L**), and disease-free survival (DSS) (Figure 1M), consistently indicated an unfavorable prognosis associated with high KRT80 expression. To further clarify whether KRT80 is an independent predictor of LUAD, regression analysis was performed in 494 samples from TCGA. Univariate Cox regression analysis suggested that KRT80 and other factors, such as T, N, and M staging, affected the prognosis of LUAD, although only the T stage was deemed an independent predictor (**Table 3**).

### The association between KRT80 and m6A-related genes, KRT80 and cuproptosis-related genes

**Figure 2:**
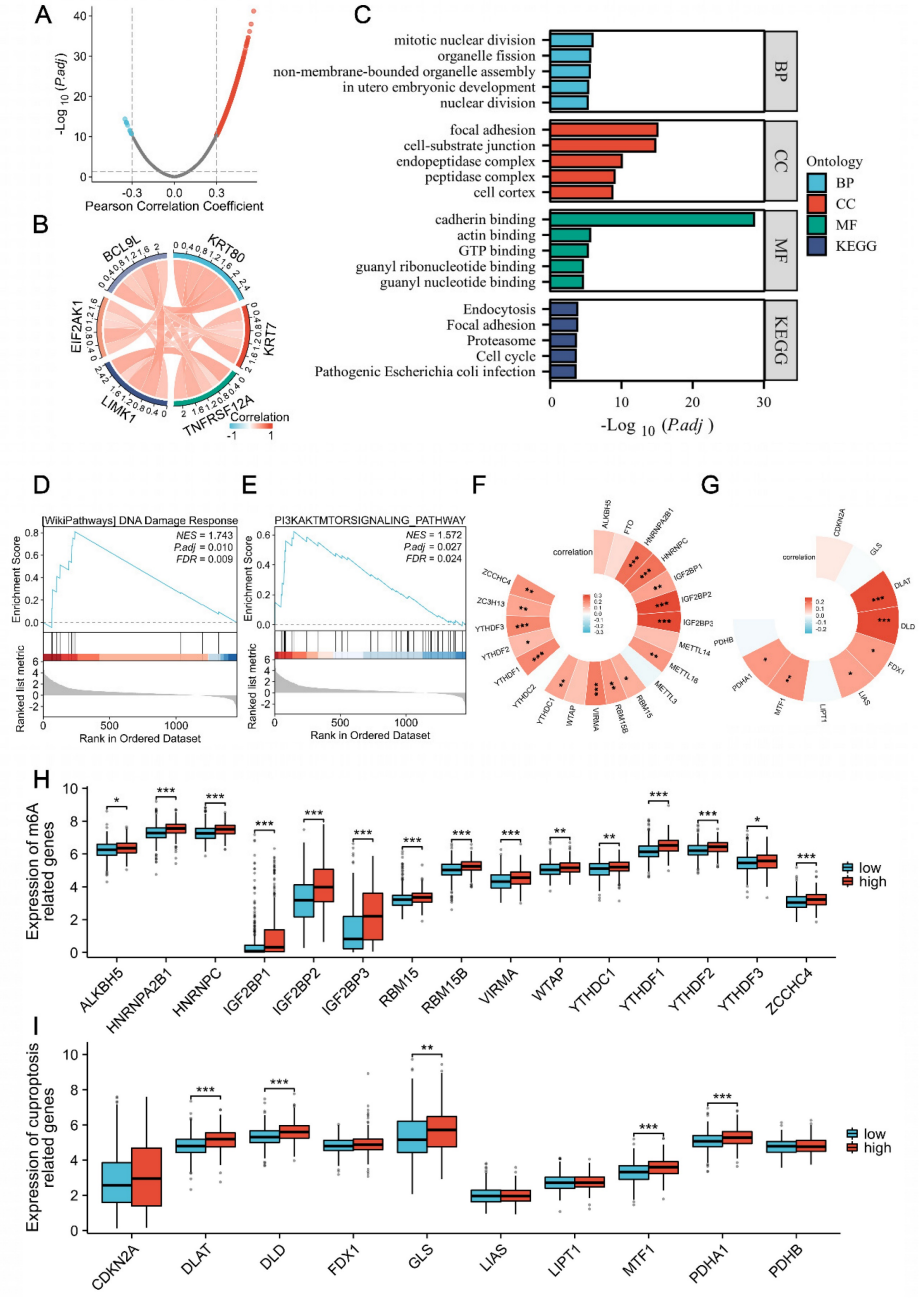
Analysis of KRT80 co-expressed genes. **(A)** Volcano plot depicting the distribution of genes co-expressed with KRT80. **(B)** Circular plot illustrating the correlation among six genes. **(C-E)** Enrichment analysis of co-expressed genes. The correlation between KRT80 and m6A-related genes **(F, H)**, as well as KRT80 and cuproptosis-related genes in TCGA** (G, I)**.

We systematically revealed the potential functions of KRT80 through correlation analysis. A volcano plot illustrated that 1,456 genes were positively correlated with KRT80 expression, while 13 genes were negatively correlated (**Figure 2A**). Among these genes, the top five associated with KRT80 were KRT7, TNFRSF12A, LIMK1, EIF2AK1, and BCL9L (**Figure 2B**). Further analysis showed that these five genes were interrelated and upregulated in LUAD, with their expression levels negatively impacting prognosis. Functional enrichment analysis revealed that co-expressed genes of KRT80 were mainly associated with cadherin binding, TP binding, cell cortex, focal adhesion, cell-substrate junction, actomyosin structure organization, actin filament bundle organization, and actin filament bundle assembly (**Figure 2C**).

KRT80-related genes were enriched in DNA damage respond and PI3K/AKT signaling Pathway (**Figure 2D, E**). M6A methylation of mRNA and cuproptosis have been associated with cancer, including LUAD. To investigate whether these processes play a role in KRT80's cancer-promoting function, we analyzed the correlation between KRT80 and 21 methylases and 10 cuproptosis-related genes. These analyses revealed significant correlations, with 15 of 21 methylase genes and 6 of 10 cuproptosis-related genes displaying significant associations with KRT80 (**Figure 2F, G**). Moreover, TCGA samples were categorized into high and low-expression groups based on average expression levels. Our analysis indicated that 16 out of 21 methylase genes were elevated in the high KRT80 expression group, and half of the 10 cuproptosis-related genes were upregulated in the high KRT80 expression group (**Figure 2H, I**).

### KRT80 elevated in LUAD tissues and cells

**Figure 3:**
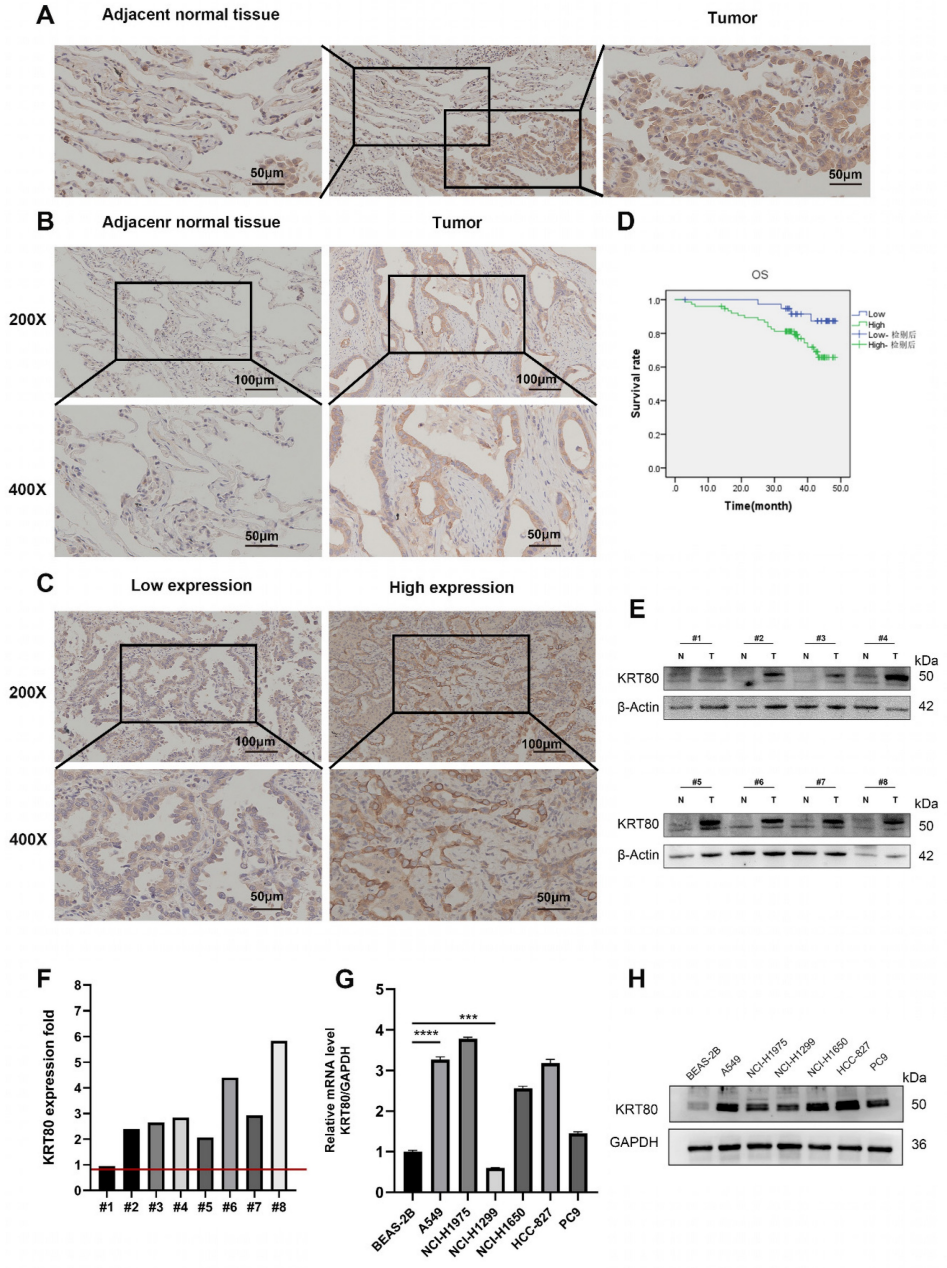
Expression of KRT80 in LUAD tissues and its prognostic value. **(A, B)** Immunohistochemical staining of KRT80 in LUAD and adjacent tissues. **(C)** Representative images showing high and low expression of KRT80 in LUAD immunohistochemical staining.** (D)** Kaplan‒Meier analysis in LUAD tissue.** (E, F)** Detection of KRT80 expression levels in lung adenocarcinoma tissues using Western Blot (WB) and quantitative PCR (qPCR). **(G, H)** Differential expression of KRT80 in six types of lung adenocarcinoma cells and lung epithelial cells.

To complement the public database transcriptome data, we conducted immunohistochemistry experiments on 115 pairs of LUAD and adjacent normal tissues. Based on staining intensity and area, these experiments confirmed the upregulation of KRT80 expression in LUAD (**Figure 3A-C**). The LUAD tissues showed higher KRT80 levels (**Figure 3E, F**), and the same results were obtained in cell lines (**Figure 3G, H**). Among them, 76 cases (66.1%) of LUAD showed high expression of KRT80, 39 cases (33.9%) showed low expression, while in adjacent tissues, 45 cases (39.1%) showed high expression of KRT80, and 70 cases (60.9%) appeared low expression (**Table 4**). Additionally, we found a positive correlation between KRT80 expression and advanced N and M stages. The log-rank test further validated the negative correlation between KRT80 expression and prognosis (**Figure 3D**). These results were consistent with TCGA database analysis (**Table 5**), supporting the conclusion that KRT80 is highly expressed in LUAD and is closely associated with prognosis, potentially serving as a diagnostic indicator for LUAD.

### KRT80 accelerates the progression of LUAD

**Figure 4:**
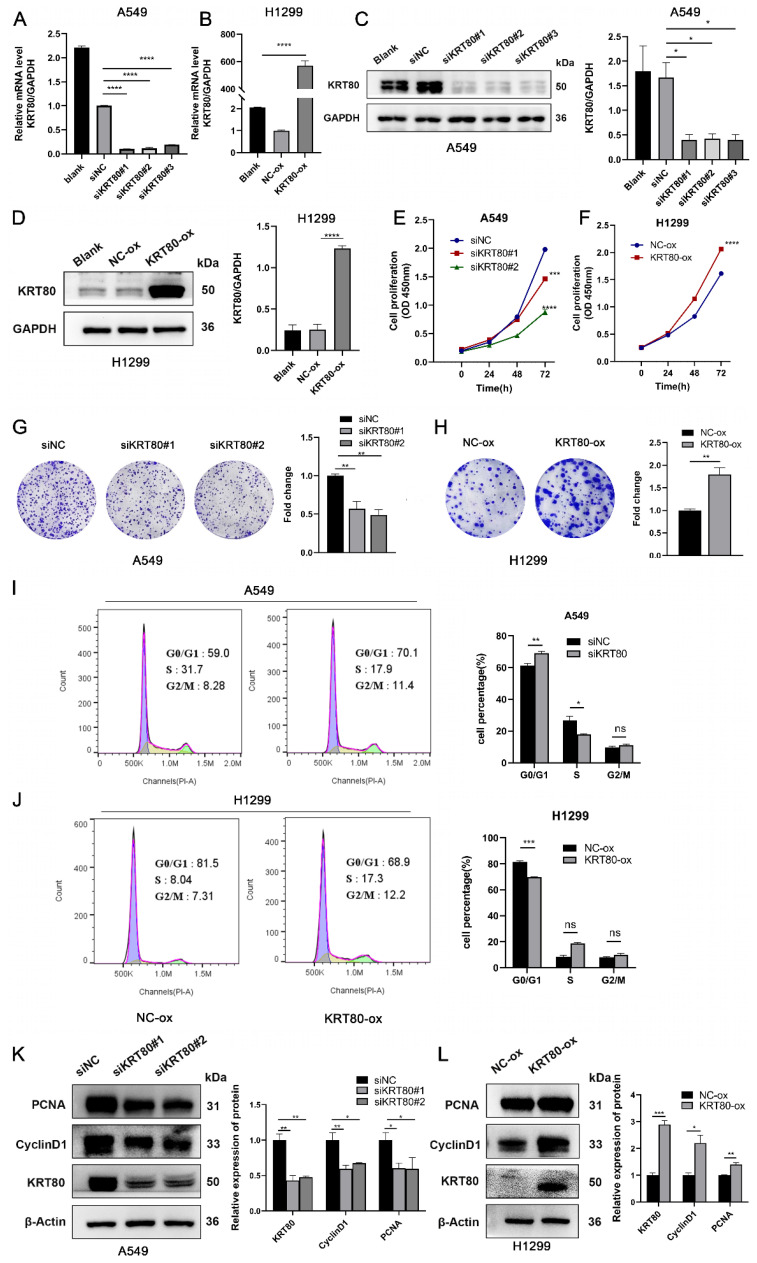
The effect of KRT80 on the proliferation of LUAD cells. **(A, B)** Real-time qRT-PCR analysis of KRT80 transfection efficiency in A549 and H1299 cells. **(C, D)** Protein level expression of KRT80 after transfection with KRT80 small interfering RNA and plasmid. **(E-H)** The impact of KRT80 on cell proliferation, as determined by Cell Counting Kit-8 (CCK8) and colony formation experiments in A549 and H1299 cells. **(I, J)** Flow cytometry analysis was performed to assess the effect of KRT80 on cell cycle regulation. (**K, L**) Western blot analysis of PCNA, CyclinD1, KRT80 expression in LUAD cells knocking down or overexpressing KRT80. *, P < 0.05; **, P < 0.01; ***, P < 0.001.

To assess the impact of KRT80 on the progression of LUAD cells, we transfected three siRNAs into A549 cells, which exhibit high KRT80 expression. Conversely, overexpression plasmids were introduced into H1299 cells with relatively low KRT80 levels. Transfection efficiency was monitored using WB and qPCR (**Figure 4A-D**). We explored the effects of KRT80 on LUAD through proliferation-related experiments. As expected, KRT80 silencing decreased OD values and cell clone counts (**Figure 4E, G**), while KRT80 overexpression led to increased OD values and cell clone counts (**Figure 4F, H**). These findings suggest a positive correlation between KRT80 and the proliferation of LUAD. Combining results from bioinformatics analysis and clinical samples, we observed that the high KRT80 group had a shorter survival period, suggesting a link between KRT80 expression and tumor progression. Then, we investigated KRT80's impact on the cell cycle. Flow cytometry analysis revealed G0/G1 cell cycle arrest in the KRT80 knockdown group compared to the control group (**Figure 4I**) Conversely, fewer H1299 cells overexpressing KRT80 were in the G0/G1 phase (**Figure 4J**). Therefore, KRT80 knockdown decreased PCNA and CyclinD1 expression (**Figure 4K**), while KRT80 overexpression increased PCNA and CyclinD1 expression (**Figure 4L**). These results indicated that knockdown of KRT80 inhibited the proliferation of LUAD cells and suppressed the transition of LUAD cells from G1 phase to S phase. Subsequently, we conducted cell migration experiments to further explore the effects of KRT80 on migration.

**Figure 5:**
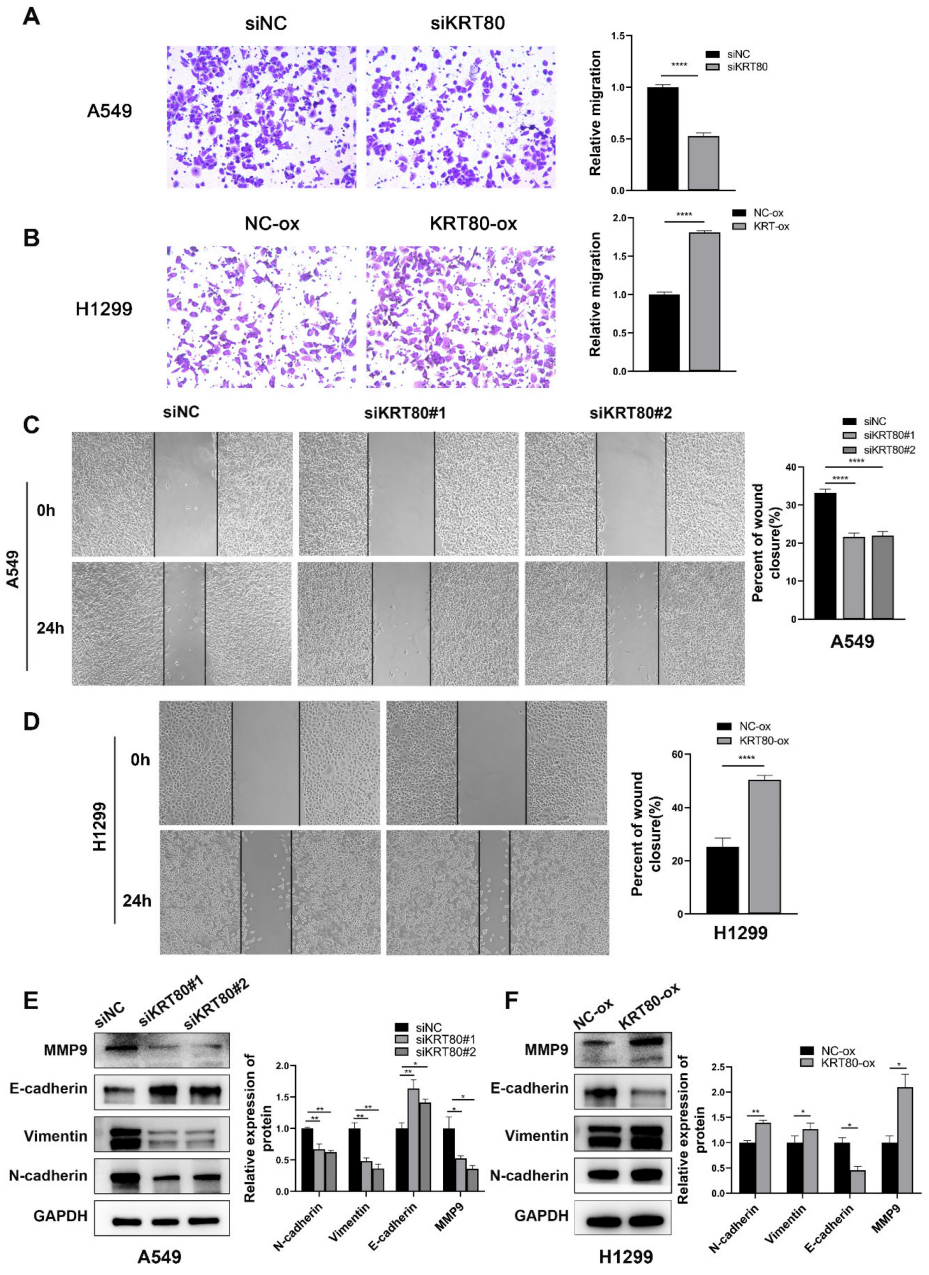
KRT80 promoted migration in LUAD cells. **(A, C)** Effect of knockdown of KRT80 expression on migration properties of A549 cells. **(B, D)** Overexpression of KRT80 in H1299 cells accelerated cell migration. **(E, F)** The protein expression of MMP9, E-cadherin, Vimentin, N-cadherin in the KRT80 knockdown or overexpression cells were determined by western blot.

The results indicated that lower KRT80 expression was associated with reduced mobility (**Figure 5A, C**), while KRT80 overexpression resulted in increased cell mobility (**Figure 5B, D**), consistent with our bioinformatics and clinical findings. Changes in KRT80 expression affected the expression levels of MMP9, N-cadherin, E-cadherin, and Vimentin, in which the expression levels of KRT80 were positively correlated with those of MMP9, N-cadherin, and Vimentin (**Figure 5E, F**).

### High expression of KRT80 activates the PI3K/AKT pathway

**Figure 6:**
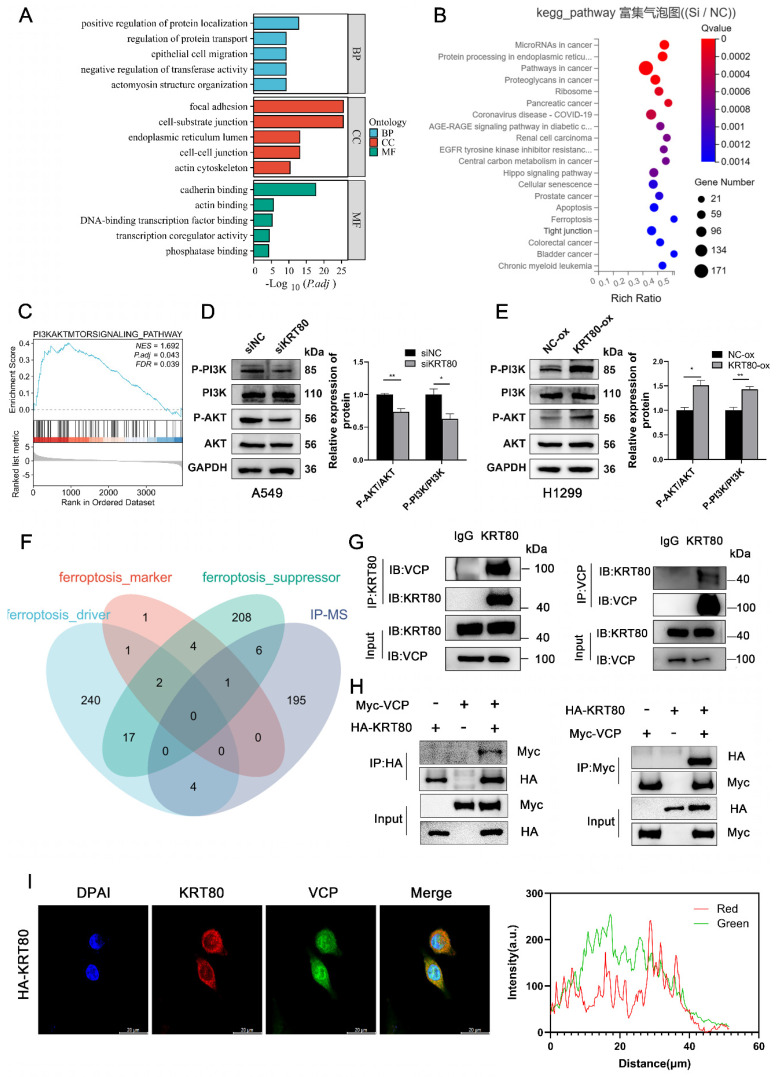
Role of knockdown or overexpression of KRT80 in signaling pathway and KRT80 interacted with VCP. **(A-C)** Enrichment analysis of differentially expressed genes. **(D, E)** Proteins extracted from cells with KRT80 knockout or KRT80 overexpression were subjected to western blotted for detecting proteins in the PI3K/AKT signaling pathway. **(F)** Venn diagram showing proteins co-precipitated by immunoprecipitation and proteins associated with ferroptosis. **(G)** Endogenous co-immunoprecipitation of KRT80 and VCP in A549 cells. **(H)** Reciprocal Exogenous co-immunoprecipitation of KRT80 and VCP in 293T cells. **(I)** Co-localization of KRT80 and VCP demonstrated by confocal microscopy.

Transcriptome sequencing was conducted in the control group and the KRT80-knockdown group to elucidate the pathways related to KRT80 in promoting the proliferation and migration of LUAD. Differentially expressed genes were identified, and functional enrichment analysis revealed a connection between these genes and transferase activity, epithelial cell migration, cell-cell junction and ferroptosis (**Figure 6A, B**). Interestingly, similar to the enrichment analysis of co-expressed genes obtained from the TCGA database, both are related to cell adhesion and connectivity. GSEA analysis demonstrated that KRT80 was enriched in the PI3K/AKT pathway, a well-established therapeutic target in lung cancer (**Figure 6C**). Accumulating evidence has indicated the relationship between the PI3K/AKT pathway and lung cancer, recognizing that this pathway is a significant therapeutic target for lung cancer 17. Western blot analysis confirmed that phosphorylated PI3K and AKT were downregulated in the KRT80 knockdown group, while total PI3K and AKT levels remained unchanged (**Figure 6D**). Conversely, in the KRT80 overexpression group, phosphorylated PI3K and AKT levels increased, while total PI3K and AKT remained unchanged, indicating that KRT80 activates the PI3K/AKT pathway (**Figure 6E**).

### Recognition of KRT80 Interacting Proteins

KRT80 has been observed to interact with PRKDC, activating the AKT signaling pathway and promoting cancer progression 9. Additional studies have suggested that OTUB2 stabilizes KRT80 by deubiquitinating it, consequently promoting gastric cancer progression 10. We conducted an IP-MS assay to identify KRT80's interacting proteins in LUAD, which yielded a list of 206 interacting proteins. Given KRT80's association with ferroptosis (**Figure 6F**), we narrowed the list by intersecting it with ferroptosis-related genes, resulting in 11 genes. Based on mass spectrometry scores, we prioritized VCP for further investigation. To validate the interaction between KRT80 and VCP, we performed a co-immunoprecipitation (co-IP) experiment, confirming the interaction. Endogenous KRT80 and VCP were successfully immunoprecipitated in A549 cells (**Figure 6G**). Moreover, exogenous KRT80 and VCP overexpressed in 293T cells exhibited a positive interaction (**Figure 6H**). Additionally, our confocal experiment confirmed the co-localization of KRT80 and VCP in A549 cells (**Figure 6I**).

**Figure 7:**
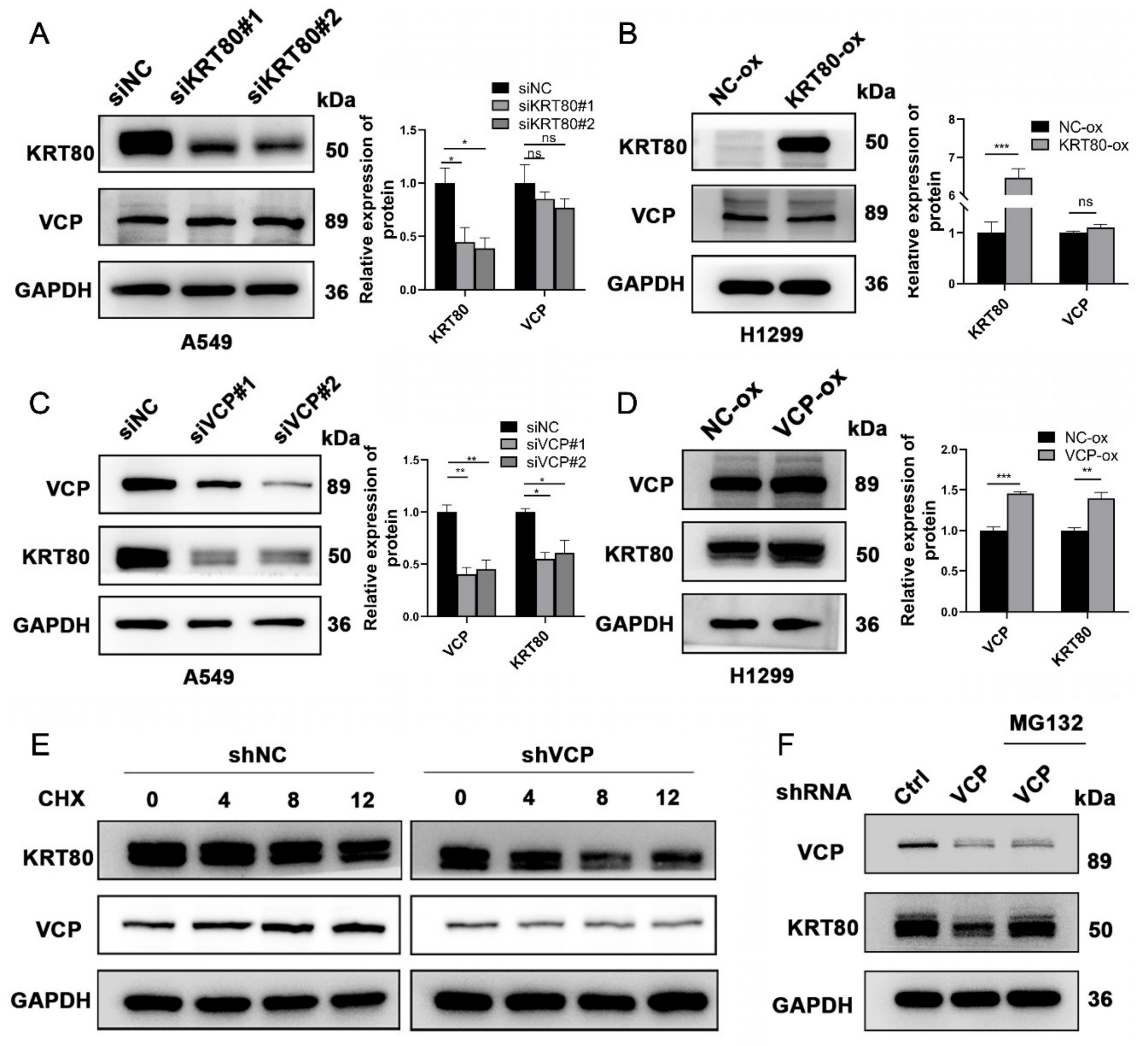
KRT80 affected the protein stability of VCP. **(A, B)** Western blot analysis of VCP protein levels upon alteration of KRT80 expression levels. **(C, D)** Western blot analysis of KRT80 protein levels when VCP is silenced or overexpressed. **(E)** Impact of VCP on KRT80 protein degradation in A549 cells. Cells stably transfected with shVCP or shNC were treated with Cycloheximide (CHX, 50 μg/mL**)** for various durations, and KRT80 protein levels were measured by Western blot. **(F)** MG132 (20 μM) was added to the shVCP cells for 10 hours.

Subsequently, we investigated the potential interplay between the expression levels of VCP and KRT80. Our findings revealed that changes in VCP expression levels were associated with a positive correlation in KRT80 expression (**Figure 7C, D**). However, when KRT80 was knocked down or overexpressed, the protein expression level of VCP remained unchanged (**Figure 7A, B**). Notably, VCP is a multifunctional protein involved in ubiquitination, and previous studies have suggested its role in enhancing the stability of the HMGB1 protein 18. To determine whether VCP affects the stability of the KRT80 protein through ubiquitination, we introduced Cycloheximide (CHX) to cell lines stably transfected with shVCP and shRNA, respectively. Interestingly, the KRT80 protein was more rapidly degraded in the shVCP group than the shRNA group, suggesting that reduced VCP expression promotes KRT80 protein degradation (**Figure 7E**). Furthermore, our experiments involving MG132 revealed that KRT80 expression decreased in the VCP knockdown group. However, after an additional 24 hours of MG132 treatment in the VCP knockdown group, KRT80 expression increased, indicating that MG132 can reverse the effect of VCP on KRT80 expression (**Figure 7F**). These findings collectively suggest that VCP interacts with KRT80, stabilizing it and implying that KRT80 is a substrate protein of VCP.

### KRT80 is essential for the cancer-promoting effects of VCP in LUAD

**Figure 8:**
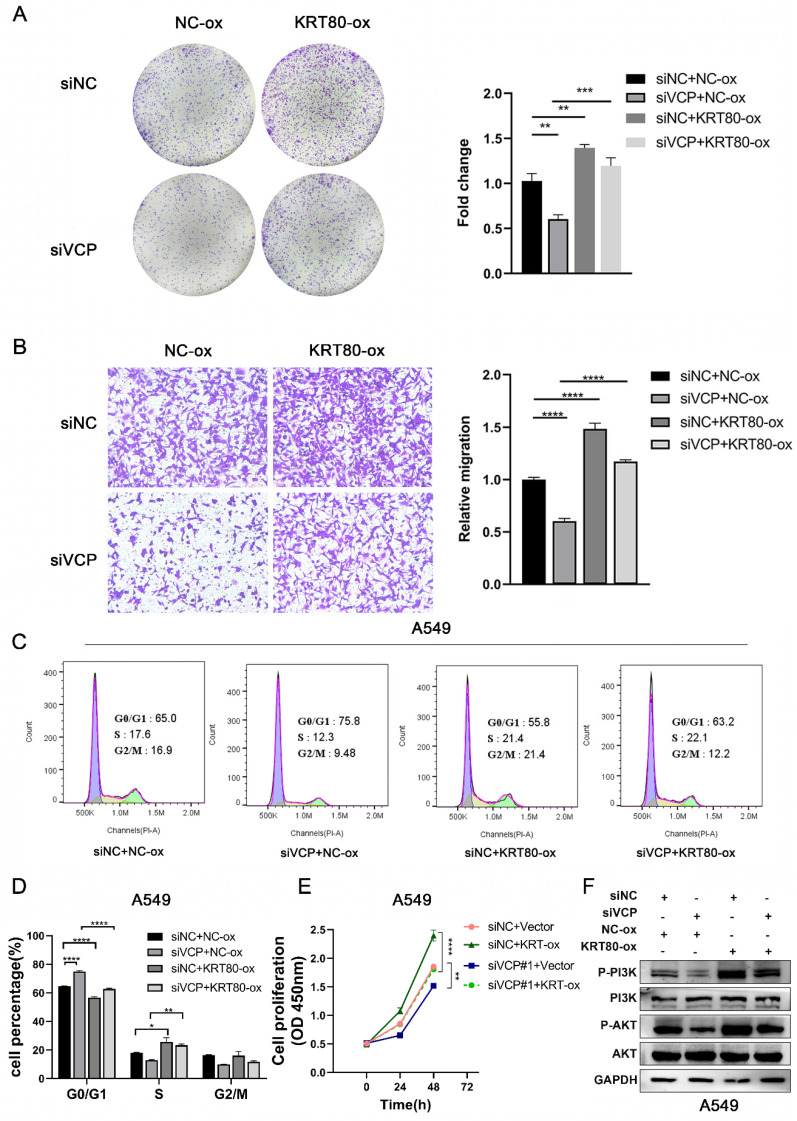
KRT80 reverses the cancer-promoting effect of VCP in LUAD. **(A-D)** CCK8 assay, colony formation experiment, migration and flow cytometry were performed to assess the effects of different treatment groups on proliferation and migration in A549 cells. **(F)** Western blot analysis of protein levels in signal pathways in different treatment groups.

To uncover the impact of KRT80 on LUAD progression induced by VCP, we assessed various aspects of cell behavior. In terms of proliferative ability, overexpression of KRT80 partially countered the inhibitory effect of VCP silencing (**Figure 8A, E**). Similar results were observed in migration experiments, where KRT80 overexpression reversed the decreased migration rate caused by VCP knockdown (**Figure 8B**). Furthermore, the proportion of cells in the G0/G1 phase was 75.8% in the VCP-transfected siRNA group, but it decreased to 63.2% in the group transfected with both siRNA and KRT80 overexpressing plasmids (**Figure 8C, D**). Additionally, the knockdown of VCP inhibited phosphorylation of AKT and PI3K, while overexpression of KRT80 partially reversed this effect (**Figure 8F**). In summary, KRT80 plays a significant role in the biological functions of VCP in promoting LUAD progression.

## Discussion

Between 2014 and 2018, the annual decline in lung cancer mortality accelerated from 2.4% to 5%, primarily attributed to the widespread use of targeted therapies 2. While highly effective EGFR and ALK tyrosine kinase inhibitors, including third-generation TKIs, have been developed, drug resistance remains a significant challenge, necessitating the ongoing search for new potential target genes 19.

Desmosomes play a critical role in tissue integrity and intercellular adhesion. The interaction between keratin and desmosomes is crucial, as it can disrupt intercellular adhesion 20. Different human type II epithelial keratins exhibit varying effects on migration in different types of tumors. Nonetheless, the roles of KRT5 and KRT8 in tumors remain controversial. KRT5 has been shown to inhibit melanoma metastasis 21, while studies have suggested that a lack of KRT5 and KRT7 can impede ovarian cancer cell migration 22. On the other hand, KRT8 has been found to promote gastric cancer progression 23. However, in lung cancer, upregulation of KRT8, induced by cisplatin, effectively countered the tumor growth-promoting effects of cancer-related fibroblasts 24. KRT7, conversely, has consistently been associated with cancer progression in various tumors, including breast cancer, colon cancer, pancreatic cancer, and gastric cancer 25-28.

Similarly, multiple reports have indicated that KRT80 promotes tumor progression. For instance, in gastric cancer, miR-671-5p and miR-4268 inhibition of KRT80 led to inactivation of the AKT signaling pathway, inhibiting tumor progression 11, 29. LINC01485 targeting miR-383-5p/KRT80 promoted colon cancer proliferation 12. In ovarian cancer, miR-206 inhibited KRT80, suppressing the MEK/ERK signaling pathway 13. Moreover, the interaction between PRKDC and KRT80 protein accelerated invasion in colon cancer 9, and OTUB2 influenced KRT80 stability through deubiquitination, promoting the malignancy of gastric cancer 10. However, comprehensive research on KRT80 in LUAD has been lacking.

Our study represents the first comprehensive analysis of KRT80's role in LUAD, integrating bioinformatics analysis, clinical tissue samples, and in vitro experiments. Bioinformatics analysis revealed elevated KRT80 expression in LUAD, confirmed in tissue samples and LUAD cells. Furthermore, upregulated KRT80 was associated with a poorer prognosis in LUAD. In vitro experiments demonstrated that KRT80 promoted the proliferation and migration of A549 and H1299 cells and impacted the cell cycle. RNA methylation, particularly m6A methylation, is an additional layer of gene expression regulation distinct from DNA methylation and histone modification. m6A methylation is the most prevalent epigenetic mRNA modification and has been implicated in the biological processes of various malignancies, including LUAD 30. Recent research has also shown that increased copper concentrations are found in samples from patients with various cancer types, including breast, lung, and gastrointestinal cancers, with copper-induced cell death, known as cuproptosis, playing a role in tumor progression 31.

We analyzed the relationship between KRT80 and m6A-related genes and KRT80 and cuproptosis-related genes, revealing that most of the 31 genes exhibited positive correlations with KRT80. This suggests that cuproptosis and m6A methylation may be involved in KRT80's promotion of LUAD progression. Enrichment analysis and western blotting demonstrated that KRT80 differential genes were enriched in the PI3K/AKT pathway. To investigate the mechanism by which KRT80 regulates the PI3K/AKT signaling pathway, we conducted an IP-MS analysis of KRT80, revealing its interaction with VCP. Furthermore, we found that KRT80 serves as a substrate of VCP. VCP is a ubiquitin-selective ATPase involved in the quality control pathways of numerous ubiquitin-dependent proteins. Previous research has indicated that VCP recognizes ubiquitination substrate proteins through specialized adapters such as UFD1, UFD2, UFD3, NPL4, UBXN6, and UBXD1^32^.

VCP is a multifunctional protein, and it has been shown to interact with MEST, promoting invasion and metastasis in lung cancer 33. Additionally, VCP recruits the E3 ligase KBTBD7 through its UBA-UBX junction to facilitate the ubiquitination of Vangl protein 34. VCP has been reported to deubiquitinate HMGB1, increasing its protein stability. Silencing HMGB1 can reverse the oncogenic effects induced by VCP 18. While VCP's role in the ubiquitin protease system is well-established, studies on VCP's interacting proteins and their ubiquitination effects remain limited. Therefore, we examined the protein stability of KRT80 and found that VCP promotes the deubiquitination of KRT80. Additionally, inhibiting KRT80 can partially counter the cancer-promoting effects of VCP in LUAD and its downstream pathway PI3K.

## Conclusion

Our research represents the first comprehensive exploration of KRT80's role and mechanisms in LUAD, incorporating bioinformatics analysis, clinical tissue samples, and in vitro experiments. Aberrant upregulation of KRT80 in LUAD is indicative of a poorer prognosis. Changes in KRT80 expression levels significantly impact LUAD progression, either activating or inactivating the PI3K/AKT pathway. Moreover, our immunoprecipitation coupled with IP-MS has revealed that KRT80 serves as a substrate protein of VCP. Interfering with KRT80 expression can influence the cancer-promoting effects driven by VCP. The interaction between KRT80 and VCP presents a promising therapeutic target in LUAD.

## Supplementary Material

Supplementary tables.

## Figures and Tables

**Table 1:** Clinical characteristics of the lung adenocarcinoma patients in TCGA

Clinical characteristics		Total (539)	Percentage (%)
Age(years)	<=65	257	47.7
	>65	263	48.8
	Unknown	19	3.5
Gender	Female	289	53.6
	Male	250	46.4
Stage	Stage I	296	54.9
	Stage II	125	23.2
	Stage III	84	15.6
	Stage IV	26	4.8
	Unknown	8	1.5
T stage	T1	176	32.7
	T2	292	54.2
	T3	49	9.1
	T4	19	3.5
	Unknown	3	0.6
N stage	N0	350	64.9
	N1	97	18.0
	N2	74	13.7
	N3	2	0.4
	Unknown	16	3.0
M stage	M0	365	67.7
	M1	26	4.8
	Unknown	148	27.5

TCGA: The Cancer Genome Atlas.

**Table 2:** Analysis of the association between KRT80 expression and clinical characteristics in TCGA.

Characteristics	Low expression of KRT80	High expression of KRT80	P value
Age, n (%)			0.220
<= 65	122 (23.5%)	135 (26%)	
> 65	139 (26.7%)	124 (23.8%)	
Gender, n (%)			0.035
Female	132 (24.5%)	157 (29.1%)	
Male	137 (25.4%)	113 (21%)	
Pathologic stage, n (%)			0.002
Stage I	163 (30.7%)	133 (25%)	
Stage II & Stage III & Stage IV	98 (18.5%)	137 (25.8%)	
Pathologic T stage, n (%)			0.023
T1	100 (18.7%)	76 (14.2%)	
T2 & T3 & T4	167 (31.2%)	193 (36%)	
Pathologic N stage, n (%)			0.001
N0	189 (36.1%)	161 (30.8%)	
N1 & N2 & N3	67 (12.8%)	106 (20.3%)	
Pathologic M stage, n (%)			0.645
M0	178 (45.6%)	187 (47.9%)	
M1	11 (2.8%)	14 (3.6%)	

**Table 3:** Univariate and multivariate Cox regression analysis in TCGA

Variable	Univariate Cox regression		multivariate Cox regression	
	HR (95%CI)	p-Value	HR (95%CI)	p-Value
Age	1.181 (0.877-1.592)	0.273		
Gender	1.081 (0.805-1.452)	0.605		
Stage	2.999 (2.196-4.095)	< 0.001	1.492 (0.775 - 2.871)	0.231
T stage	2.234 (1.303-3.831)	0.003	1.631 (1.029 - 2.586)	0.037
N stage	2.633 (1.951-3.553)	< 0.001	1.803 (0.987 - 3.296)	0.055
M stage	1.707 (1.204-2.422)	0.003	1.575 (0.847 - 2.927)	0.151
riskScore	1.538 (1.141-2.072)	0.005	1.138 (0.806 - 1.608)	0.461

**Table 4:** Expression of KRT80 protein in LUAD tissues and normal tissues

Sample	n	KRT80		OR (95%CI)	p-Value
		Low expression (%)	High expression (%)		
Normal	115	70 (60.9)	45 (39.1)	Ref	
Cancer	115	39 (33.9)	76 (66.1)	3.031 (1.770-5.191)	<0.001

**Table 5:** Logistic analysis of the association between KRT80 expression and clinical characteristics.

Characteristic		KRT80 (n=115)		OR (95%CI)	p-Value
		Low expression	High expression		
Age (years)	≤60	16	36	Ref	
	>60	23	40	0.594 (0.237-1.492)	0.268
Gender	Female	17	28	Ref	
	male	22	48	1.237 (0.515-2.970)	0.634
T stage	T1	24	29	Ref	
	T2-4	15	47	2.271 (0.922-5.597)	0.075
N stage	N0	32	39	Ref	
	N1-3	7	37	3.716 (1.385-9.968)	0.009
M stage	M0	33	44	Ref	
	M1	6	32	3.127 (1.106-8.841)	0.032

## Abbreviations

KRT80: Keratin 80
VCP: Valosin-containing protein
LUAD: lung adenocarcinoma
IFs: intermediate filaments
UBX: ubiquitin regulated X
CHX: Cycloheximide
